# *Weizmannia coagulans* BC99 Improve Cognitive Impairment Induced by Chronic Sleep Deprivation via Inhibiting the Brain and Intestine’s *NLRP3* Inflammasome

**DOI:** 10.3390/foods14060989

**Published:** 2025-03-14

**Authors:** Qiaoqiao Sun, Jiajia Fan, Lina Zhao, Zhen Qu, Yao Dong, Ying Wu, Shaobin Gu

**Affiliations:** 1 College of Food and Bioengineering, Henan University of Science and Technology, Luoyang 471000, China; 19837952968@163.com (Q.S.); 13290501131@163.com (J.F.); zhaolina@haust.edu.cn (L.Z.); q819707110@163.com (Z.Q.); 2 Henan Engineering Research Center of Food Material, Henan University of Science and Technology, Luoyang 471023, China; 3 Germline Stem Cells and Microenvironment Lab, College of Animal Science and Technology, Nanjing Agricultural University, Nanjing 210095, China; florady0327@163.com

**Keywords:** chronic sleep disorder, cognitive deficits, *W. coagulans*, inflammation, *NLRP3* signaling pathway

## Abstract

*Weizmannia coagulans* BC99, a Gram-positive, spore-forming, lactic acid-producing bacterium is renowned for its resilience and health-promoting properties, *W. coagulans* BC99 survives harsh environments, including high temperatures and gastric acidity, enabling effective delivery to the intestines. The consequences of chronic sleep deprivation (SD) include memory deficits and gastrointestinal dysfunction. In this study, a chronic sleep deprivation cognitive impairment model was established by using a sleep deprivation instrument and *W. coagulans* BC99 was given by gavage for 4 weeks to explore the mechanism by which BC99 improves cognitive impairment in sleep-deprived mice. BC99 improved cognitive abnormalities in novel object recognition tests induced by chronic sleep deprivation and showed behavior related to spatial memory in the Morris water maze test. *W. coagulans* BC99 reduced the heart mass index of sleep-deprived mice, increased the sleep-related neurotransmitters 5-HT and DA, decreased corticosterone and norepinephrine, and increased alpha diversity and community similarity. It reduced the abundance of harmful bacteria such as *Olsenella*, increased the abundance of beneficial bacteria such as *Lactobacillus* and *Bifidobacterium*, and promoted the production of short-chain fatty acids (SCFAs). *W. coagulans* BC99 also inhibits LPS translocation and the elevation of peripheral inflammatory factors by maintaining the integrity of the intestinal barrier and inhibiting the expression of the *NLRP3* signaling pathway in the jejunum, thereby inhibiting the *NLRP3* inflammasome in the brain of mice and reducing inflammatory factors in the brain, providing a favorable environment for the recovery of cognitive function. The present study confirmed that *W. coagulans* BC99 ameliorated cognitive impairment in chronic sleep-deprived mice by improving gut microbiota, especially by promoting SCFAs production and inhibiting the *NLRP3* signaling pathway in the jejunum and brain. These findings may help guide the treatment of insomnia or other sleep disorders through dietary strategies.

## 1. Introduction

According to the World Health Organization, over one-third of the world’s population suffers from insufficient sleep. In China, the proportion is as high as 38.2%, with more than 300 million people suffering from different degrees of sleep disorders. There is increasing evidence that SD may cause cognitive degradation, including memory impairment and cognitive decline, and even increase the risk of dementia. Insomnia is a predictor of the onset of mental disorders [1,2]. The main pharmacologic treatment option for insomnia is psychotropic medications, such as benzodiazepines (BZs). Long-term use of BZs can negatively affect cognitive function and lead to potential drug dependence, so new ways to treat insomnia are needed. As an important dietary supplement regulating gut microbiota, probiotics have shown great potential in regulating brain function, such as improving neurotransmitter biosynthesis, blood–brain integrity, and myelination. One study showed that *Bifidobacterium breve CCFM1025* attenuated cognitive behavioral abnormalities induced by sleep deprivation. Another study used *Lactobacillus plantarum Lp-115*, *Lactobacillus paracaseus Lpc-37*, and *Bifidobacterium animalis* subsp. These probiotics significantly improved recognition memory deficits, spatial working memory deficits, and contextual long-term memory impairment in sleep-deprived mice upon novel object recognition tests. *Lacticaseibacillus rhamnosus GG* (LGG) has also been found to help improve gut and neuroinflammation caused by sleep deprivation. *Akkermansia muciniphila (Akk)* can improve cognitive impairment induced by sleep deprivation, indicating that insomnia and sleep restriction induce significant structural and functional changes in these gut microbiota. However, there are few studies on the role of *W. coagulans* BC99 in cognitive impairment induced by sleep deprivation.

Insomnia and sleep restriction have been reported to cause significant structural and functional changes in the gut microbiota [3,4]. Alterations in the gut microbiome have been implicated in the pathophysiology of psychiatric disorders such as autism spectrum disorders [5], depression [6,7], and schizophrenia [8], suggesting that the gut microbiome influences multiple aspects of neuroendocrine function and brain development. Gut microbiota can produce a large number of bioactive substances, such as neurotransmitters, amino acids, short-chain fatty acids, unsaturated fatty acids, etc. Short-chain fatty acids (SCFAs), including acetic acid, propionic acid, and butyric acid, can enter the circulation system, pass the blood–brain barrier, and transmit signals to the brain. Neurotransmitters, including acetylcholine, norepinephrine, and γ-aminobutyric acid (GABA), are synthesized by intestinal flora and affect brain function through the gut–brain axis. Intestinal flora also affects the production of pro-inflammatory and anti-inflammatory factors and exerts pro-inflammatory and anti-inflammatory immune effects on the brain through the circulatory system [9]. These gut microbiota have also been viewed as important factors affecting memory performance in mice. Intestinal microbial metabolites, such as short-chain fatty acids (SCFAs) [10], are mainly produced in the large intestine through anaerobic bacterial fermentation to maintain intestinal immune function and regulate intestinal barrier function [11]. In macrophages, SCFAs inhibit lipopolysaccharide (LPS) and cytokine-stimulated pro-inflammatory mediators, including tumor necrosis factor-α (TNF-α) and interleukin-6 (IL-6) [12]. SCFAs have also been shown to regulate microglia maturation and function, with potential benefits in the prevention of neuroinflammation processes. In addition, SCFAs have been hypothesized to play a mediating role in the microbial–gut–brain axis [13]. Emerging evidence suggests that gut microbiota can affect intestinal barrier permeability, increase the leakage of intestinal endotoxin, and activate Toll-like receptor 4 (TLR4), eventually leading to dysregulation of the inflammatory response [14]. Microbial dysbiosis caused by chronic sleep disruption may also contribute to the development of systemic inflammation and insulin resistance in mice [15]. These results suggest that the microbiota–gut–inflammation–brain axis plays an important role in the mechanism of cognitive decline caused by SD.

*NLRP3* is the most characteristic inflammasome, containing the sensor (NLRP3), adaptor protein *ASC* (apoptosis-associated speck-like protein including caspase activation and recruitment domain), and effector protein (cleaved caspase-1), whose activation initiates downstream inflammatory cascades and forms peripheral and central immune/inflammatory responses. Of note, a growing body of research supports a critical role for the *NLRP3* inflammasome complex in the neuroinflammation observed in AD (Alzheimer’s disease), because its activation in microglia triggers the secretion of pro-inflammatory cytokines interleukin (IL)-18 and Il-1β by activating cysteine-containing aspartate-specific proteinase 1 (cleaved caspase-1), which ultimately exacerbates the pathological progression of AD and induces synaptic loss, neuronal necrosis, and apoptosis. In addition, previous studies have reported that the *NLRP3* inflammasome is activated in the gut of mouse models of many diseases and is involved in regulating intestinal homeostasis. Bacterial products can represent the first and second signals that activate the *NLRP3* inflammasome. It is reasonable to assume that the *NLRP3* inflammasome is at the crossroads of microbiota–gut–inflammation–brain communication. However, to date, there is no direct causal relationship between gut microbiota alterations, *NLRP3* activation, and brain pathology in chronic SD. We hypothesized that compositions after chronic SD could disrupt the intestinal mucosal barrier by activating the *NLRP3* inflammasome in the gut and initiate intestinal and peripheral inflammatory responses, which, in turn, could lead to *NLRP3*-controlled neuroinflammation in the central nervous system (CNS) and ultimately lead to cognitive impairment. SD is becoming more severe in modern society and may be a treatable risk factor for AD.

These studies suggest that gut microbiota may mediate the deleterious effects of sleep loss. This study aims to investigate the role and mechanism of *W. coagulans* BC99 in a mouse model of cognitive impairment induced by sleep deprivation. Behavioral, serological, and ELISA tests were performed to assess cognition and inflammation. 16S rRNA gene sequencing was used to test the effect of SD on the intestinal microbiota of mice. The *NLRP3* inflammatory signaling pathway was tested by qPCR. In this study, we hypothesized that *W. coagulans* BC99 could improve cognitive impairment induced by chronic SD by ameliorating intestinal flora and metabolic dysregulation, inhibiting LPS translocation, and subsequently inhibiting *NLRP3* inflammatory signaling pathway activation in the central nervous system. This study is not only expected to provide new insights into the complex relationship between gut microbiota and cognitive function but also may provide a scientific basis for the development of new treatment strategies for sleep disorders related to cognitive impairment, which has important theoretical significance and clinical application value.

## 2. Materials and Methods

### 2.1. Experimental Methods

#### Preparation of Probiotic Suspension

**Figure 1:**
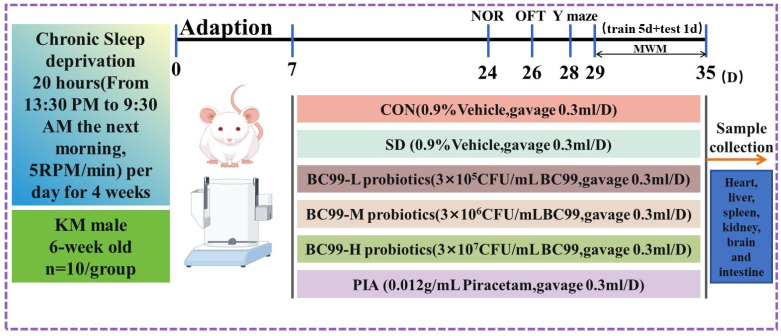
Experiment timeline.

The lowest effective dose of probiotics is usually 10^7^ CFU/mL [16]. In this study, 0.3 g of *W. coagulans* [17] BC99 powder was weighed accurately in 10 mL of 0.90% sterile normal saline to obtain 3 × 10^7^ CFU/mL bacterial suspension and then diluted in sterile normal saline to obtain 3 × 10^6^ and 3 × 10^5^ CFU/mL bacterial suspension. Additionally, 0.1224 g 98% piracetam was weighed in 10 mL 0.90% sterile saline to obtain 0.012 g/mL drug suspension. After preparation, the drug suspension was refrigerated at 4 °C for later use. After the test, the mice were fasted for 12 h and sacrificed. Serum and brain tissue samples were collected to determine and analyze related indicators. The contents and processes of the experiment were planned according to international and national ethical standards for biomedical research. The experimental design is shown in Figure 1.

### 2.2. Design of Animal Experiments

The Animal Welfare Ethics Committee of Henan University of Science and Technology (Luoyang, China) approved the experimental procedures. The committee’s approval certification ID is 4103050048305. The license number for the use of experimental animals is SYXK(Yu)2021-0020. Sixty healthy Kunming male mice (6 weeks old) weighing (30 ± 2) g were purchased from Zhengzhou University (Zhengzhou, China) and kept on a 12 h light/12 h night alternating cycle. The animals were housed at a constant temperature of (22 ± 2) °C and a humidity of (50 ± 5)%. Sixty mice were randomly assigned to six groups (n = 10 each): the control (CON) group, the chronic sleep deprivation (SD) group, the chronic sleep deprivation +3 × 10^5^ CFU/mL BC99 (BC99-L) group, the chronic sleep deprivation +3 × 10^6^ CFU/mL BC99 (BC99-M) group, the chronic sleep deprivation +3 × 10^7^ CFU/mL BC99 (BC99-H) group, and the chronic sleep deprivation + piracetam (PIA) group. The control group and the model group were treated with 0.3 mL of 0.90% normal saline, and the low-, medium-, and high-dose groups were treated with 0.3 mL of 3 × 10^5^, 3 × 10^6^, and 3 × 10^7^ CFU/mL bacterial suspension by intragastric administration, respectively, for 4 weeks. After the test, the mice were fasted for 12 h and sacrificed. Serum and brain tissue samples were collected to determine and analyze related indicators. 

### 2.3. Chronic Sleep Deprivation

In the second week, mice were deprived of 20 h of sleep for 28 consecutive days using a novel chronic sleep deprivation meter [18] at 5 rpm. Chronic sleep deprivation was performed for 20 h from 13:30 to 09:30 the next day, and all mice were returned to their cages for 4 h from 09:30 to 13:30 the next day. To establish a mouse model of cognitive impairment induced by chronic sleep deprivation. Control mice were kept in the same cages as those used for the mice subjected to experimental conditions. Food and water were provided equally. Compared with the normal control group, the mice were more sedentary and showed increased daytime activity, laziness at night, decreased body weight, slow reactions, and, sometimes, anxiety and irritability, which was significantly different, indicating that the model was successfully prepared.

### 2.4. Probiotic Therapy

After 7 days of adaptive feeding, the mice were randomly divided into a control group; a model group; *W. coagulans* BC99 low-, medium-, and high-dose groups; and a piracetam group. Then, the mice in each group were subjected to the following experiments: the control group and the model group were gavaged with 0.90% sterile saline 0.3 mL daily, and the low-, medium-, and high-dose groups were gavaged with 3 × 10^5^, 3 × 10^6^ and 3 × 10^7^ CFU/mL bacterial suspension 0.3 mL daily for 4 weeks.

### 2.5. Morris Water Maze (MWM) Test

As a relatively objective experiment to study and evaluate the learning and memory function of rodents, water maze has been widely used in the study of psychological diseases such as depression and anxiety and neurodegenerative diseases such as Alzheimer’s disease and Parkinson’s disease [19]. Based on previous research, our test was outlined as follows: The water maze used was 1.2 m in diameter and 35 cm in height, and paper signs of different colors and shapes were posted on shelves around the test pool. The test pool was divided into four quadrants. On the day before the training trial (day 0), a small platform was placed 2 cm above the water. Mice were adapted to each quadrant of the maze for 1 min each, and during the subsequent 5-day learning trials (days 1–5), the water in the pool was mixed with black ink, and the platform was submerged 1.5 cm below the surface.

During the learning trial, each mouse was given 1 min to find the platform location, and if it failed, it was guided to the platform location, before remaining on the platform for 10 s and being trained to locate the target platform for 1 min. Each mouse was given four trials daily, starting at four locations around the pool, with a 25 min interval between rounds. On day 6, the escape platform was removed, the mice were placed in water from the quadrant (second quadrant) contralateral to the quadrant where the previous platform was located (fourth quadrant), and the mice were given 60 s to search for the maze in the trial. The time taken to reach the target platform (escape latency), the time taken to cross the target platform, and the number of platform quadrants were recorded throughout the trial. Data were recorded by an intelligent video tracking system for further analysis.

### 2.6. Novel Object Recognition Test

The novel object recognition (NOR) test assesses cognitive ability [20]. One day before the experiment, mice were placed in two 40 cm × 40 cm × 40 cm square cardboard boxes with the same cube placed at the two corners, and the mice were allowed to adapt to the environment for 24 h. Subsequently, mice were reintroduced to the pen, and a randomly selected trigonophore replaced the previously familiar cube. The animals were allowed to explore the environment for 6 min. During that time, their interactions were recorded using TAM’s Open Field test (OFT) software (Smart 3.0) to quantify time spent with each object, expressed as a percentage of time spent interacting with each object. The cognitive index was calculated using the following formula: t novel/(t novel + t familiar). The discrimination index was calculated using the following formula: (t novel − t familiar)/(t novel + t familiar).

### 2.7. Measurement of Plasma Inflammatory Response

Intraocular blood samples were collected before the mice were sacrificed and centrifuged at 4000 RPM for 15 min at 4 °C to promote plasma production. IL-6, IL-10, and TNF-α concentrations in plasma samples were quantified per the guidelines provided with the ELISA kit (Shanghai Hepai Biotechnology Co., Ltd., Shanghai, China).

### 2.8. Enzyme-Linked Immunosorbent Assay

The enzyme-linked immunosorbent assay (ELISA) kit provided by Shanghai Hepai Biotechnology Co., Ltd. (Shanghai, China) was used in this study according to the protocol listed by the manufacturer. Plasma levels of several hormones and neurotransmitters, including corticosteroids (CORT), norepinephrine (NE), serotonin (5-HT), dopamine (DA), and lipopolysaccharide (LPS), were quantified. For tissue homogenization, frozen hippocampal tissue was extracted from a refrigerator at −80 °C and precisely weighed to 0.025 g; it was mixed in a 1:9 ratio with 0.9% normal saline (e.g., 1 g of tissue is often mixed with 9 mL of normal saline). Absorbance was then measured at 450 nm using a microplate reader.

### 2.9. Assessment of Oxidative Stress in Brain Tissue

To detect oxidative stress, 0.025 g brain tissue samples were weighed and mixed with 0.25 mL of the extract. The mixture was mixed in an ice water bath with a homogenizer, followed by centrifugation at 3000 r/min for 10 min at 4 °C. The supernatant was pooled at once into a 1.5 mL centrifuge tube to obtain tissue homogenates, and the concentrations of GSH and MDA in the homogenates were determined according to the kit’s instruction manual.

### 2.10. Measurement of Fecal SCFAs

Gas chromatography–mass spectrometry (GC-MS) was used to analyze the short-chain fatty acids (SCFAs) in frozen fecal samples. Moreover, 0.20 g of feces was mixed with 1.60 mL sterilized deionized water. The mixture was mixed evenly and shaken, left at room temperature for 20 min, and centrifuged at 15,000 rpm for 15 min at 4 °C, and the supernatant was transferred to a new EP tube. The fecal precipitate was added with 1.6 mL sterilized deionized water again, and the above operation was repeated. After filtering through a 0.22 μm filter, 0.20 mL of supernatant, 0.70 mL of sterile water, and 0.10 mL of 100 μg/mL n-butyl alcohol were mixed for GC detection. Detection was performed using a GC-MS system (TSQ 9000, Thermo Scientific, Waltham, MA, USA) equipped with an Rtx-WAX capillary column, HP-5 column, injection temperature of 250 °C, column temperature of 40 °C, ion source temperature of 220 °C, and transmission line temperature of 250 °C. The sample volume was 1 μL. The external standard method was used to draw standard curves for acetic acid, propionic acid, and butyric acid, and samples were quantified according to peak areas.

### 2.11. Real-Time Fluorescence Quantitative PCR

RNA was extracted from mouse tissues using an AG kit (AG21101, AG, Changsha, China) and subsequently reverse-transcribed using the PrimeScript RT package (Cat, ABC Clone RK 20429, Wuhan, China). Quantitative PCR (qPCR) was performed using SYBR Green Master Mix (Cat, RK21203, AB Clone, Wuhan, China). Intact RNA was extracted from jejunal and brain tissues using a steady-state pure RNA extraction kit according to the manufacturer’s protocol, and cDNA synthesis was performed using 5× Evo M-MLV RT Master Mix (Accurate Biology, Changsha, China). The primer sequences used in the experiments are shown in Table 1. Real-time PCR was completed using qPCR SYBR Green Pro Taq Hs Premix (Accurate Biology, Changsha, China) under the following thermal cycling conditions: initial denaturation at 95 °C for 30 s, followed by 40 cycles of denaturation at 95 °C for 5 s and annealing/extension at 60 °C for 30 s. The mRNA expression levels of *NLRP3*, *ASC*, *Caspase-1*, *Occludin*, and *ZO-1* were detected by real-time fluorescence quantitative PCR, and for each group, the process was repeated 5 times. The relative expression range of β-actin was determined by the 2^−△△Ct^ method using β-actin as an internal management gene.

### 2.12. 16S rRNA Microbiota Assessment and Bioinformatics

DNA was extracted from sparkling cecal samples. The initial sequencing data, known as raw data, were spliced according to overlapping regions and visualized by removing low-quality and chimeric sequences, resulting in extraordinarily clean data. Subsequently, we performed sequence noise discounting using QIIME2 DADA2 to eliminate possible PCR artifacts in the high-throughput sequencing data. These sequences were similarly complex and processed entirely based on clean data to eliminate any closed PCR amplification and sequencing errors, resulting in consultant biological sequences known as Amplicon Sequence Variants (ASVs) and their corresponding abundance tables. Subsequent alpha diversity analyses, beta diversity analyses, species composition and divergence analyses, and functional composition were further performed. Linear discriminant analysis (LDA) effect size (LEfSe) was calculated to distinguish microbial taxa (*p* < 0.05, lg LDA > 3.0 as the threshold). Functional prediction was performed using PICRUSt2 (Biotree, Shanghai, China).

### 2.13. Statistical Analysis

In this experiment, OriginPro2024 software was used for mapping. The experimental data were expressed as mean ± standard error, and SPSS 26.0 software was used for one-way ANOVA or Kruskal–Wallis statistical analysis, where *p* < 0.05 was considered statistically significant.

## 3. Results

### 3.1. Effects of W. coagulans BC99 on Behavioral and Physiological Indices of Chronic Sleep Deprivation Mice

**Figure 2:**
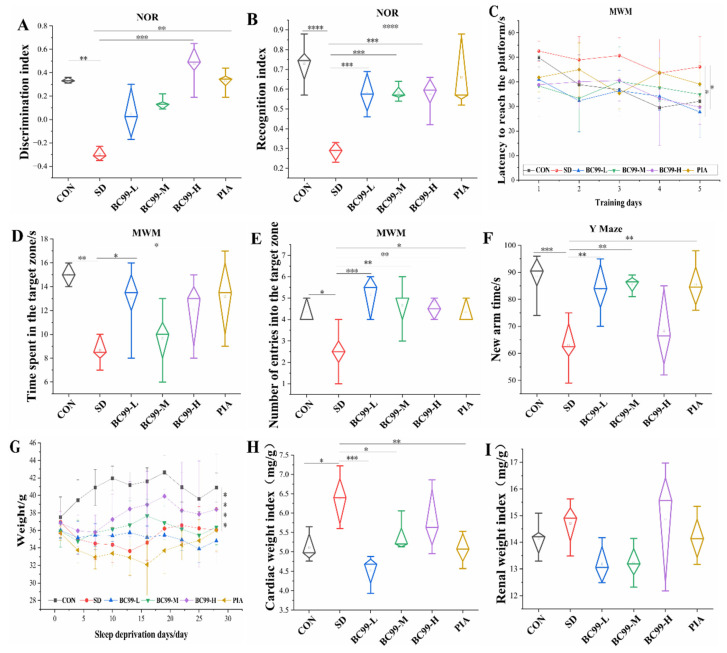
Effects of BC99 on behavioral and physiological indices of chronic sleep deprivation mice (n = 6). (**A**) Discrimination index. (**B**) Recognition index. (**C**) Latency to reach the platform. (**D**) Time spent in the target zone. (**E**) Number of entries into the target zone. (**F**) New arm time. (**G**) Weight. (**H**) Cardiac weight index. (**I**) Renal weight index. Note: NOR—new object recognition experiment; MWM—Morris water maze experiment. * *p* < 0.05, ** *p* < 0.01, *** *p* < 0.001, and **** *p* < 0.001.

The novel object recognition (NOR) test is shown in Figure 2A,B. As shown in Figure 2A, after a period of chronic sleep deprivation, the discrimination index of the mice decreased significantly (*p* < 0.01), while as shown in Figure 2B, the cognitive index of the mice decreased significantly (*p* < 0.001). On the contrary, the cognitive index test of low-, medium- and high-dose *W. coagulans* BC99-treated mice showed a considerable improvement (1.68-fold increase), which was significantly higher than that of the model group (*p* < 0.001). In addition, compared with the model group, the discrimination index of the high-dose probiotics group was significantly improved (by 2.42 times, *p* < 0.001), and the discrimination index of the piracetam treatment group was also significantly improved (*p* < 0.01). These results suggest that both *W. coagulans* BC99 and piracetam are effective in improving cognitive deficits induced by chronic sleep deprivation. After 4 weeks of chronic sleep deprivation, the results of the Morris water maze test (continuous training for 5 days to find the platform) showed that the platform-finding latency of the model group mice on the 5th day (Figure 2C) was higher than that of the control group (*p* = 0.10), indicating that the memory ability of the chronic sleep deprivation group mice was decreased. However, both low and high doses of *W. coagulans* BC99 significantly reduced the platform-finding latency in mice, indicating that BC99 reversed the impaired spatial memory ability in mice. In addition, the time spent in the target quadrant and the number of crossings were significantly reduced in the model group compared with the control group (*p* < 0.01), highlighting the deleterious effects of chronic sleep deprivation on spatial memory. In contrast, both low-dose (0.96-fold improvement) and high-dose (1.09-fold improvement) *W. coagulans* BC99 treatment significantly improved the time to cross the target quadrant (Figure 2D) compared with the model group (both *p* < 0.05), as shown in Figure 2E, and low-dose BC99 treatment greatly increased the number of target quadrant crossings. It increased by 2.59 times (*p* < 0.001). Similarly, the *W. coagulans* BC99 medium- and high-dose groups also showed a significant increase in the number of target quadrant crossings (*p* < 0.01), and the BC99 treatment group also showed a significant increase in this index (*p* < 0.05). This study confirmed that *W. coagulans* BC99 enhanced learning and memory in sleep-deprived mice. The results of the Y-maze, shown in Figure 2F, show that the time to enter the novel arm was greatly reduced in the model group (*p* < 0.001). In contrast, low- and medium-dose *W. coagulans* BC99 and piracetam treatment significantly increased the time to entry into the novel arm (all *p* < 0.01). In conclusion, *W. coagulans* BC99 can improve cognitive impairment in chronic sleep-deprived mice.

Figure 2G provides the changes in body weight in the six groups of mice. On days 1, 4, 7, 10, 13, 16, 19, 22, 25, and 28, the body weight of chronic sleep deprivation mice was significantly lower than that of control mice (*p* < 0.001), indicating that chronic sleep deprivation significantly hindered the body weight growth of mice. In addition, *W. coagulans* BC99 treatment appeared to counteract weight loss in the chronic sleep deprivation group. Compared with the control group, the heart weight index (Figure 2H) of the model group was significantly increased (*p* < 0.05), and the kidney weight index (Figure 2I) of the model group was not significantly increased (*p* > 0.05), indicating that chronic sleep deprivation had little effect on the kidneys of the mice, and chronic sleep deprivation had a great effect on the hearts of the mice, indicating that chronic sleep deprivation led to increased energy expenditure in the mice. The body’s metabolism is hyperactive. The heart weight index of the chronic sleep deprivation group was significantly lower than that of the chronic sleep deprivation group (*p* < 0.001), and the heart weight index of the medium-dose *W. coagulans* BC99-protected mice was significantly higher than that of the model group (*p* < 0.05). Compared with the model group, the heart weight index of the piracetam treatment group was significantly lower than that of the chronic sleep deprivation group (*p* < 0.01). BC99-protected mice and piracetam-treated mice showed a non-significant reduction in kidney weight index, indicating that *W. coagulans* BC99 ameliorated the metabolic abnormalities caused by sleep stripping.

### 3.2. Effect of W. coagulans BC99 on Biochemical Indices in Chronic Sleep Deprivation Mice

**Figure 3:**
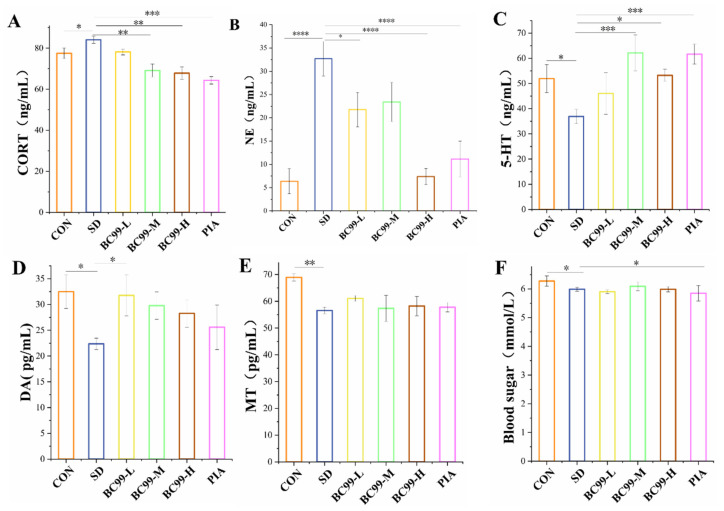
Effect of BC99 on biochemical indices in chronic sleep deprivation mice (n = 3). (**A**) The level of CORT in the plasma. (**B**) The level of NE in the plasma. (**C**) The level of 5-HT in the hippocampus. (**D**) The level of DA in the hippocampus. (**E**) The level of MT in the plasma. (**F**) The level of blood sugar in the plasma. Note: CORT—corticosterone, NE—norepinephrine, 5-HT—5-Hydroxytryptamine, DA—dopamine, and MT—melatonin. * *p* < 0.05, ** *p* < 0.01, *** *p* < 0.001, and **** *p* < 0.001.

Corticosterone content (Figure 3A) was significantly increased in the model group compared with the control group (*p* < 0.05), reduced considerably in the medium-dose *W. coagulans* BC99 group compared with the model group (*p* < 0.01), and greatly reduced in the high-dose *W. coagulans* BC99 and administration groups (*p* < 0.001). The above results suggest that chronic sleep deprivation promotes the HPA axis and increases corticosterone release, while *W. coagulans* BC99 and piracetam treat the HPA axis abnormalities. Norepinephrine (NE) is a monoamine neurotransmitter involved in a range of neurobehavioral processes, such as learning, memory, and addiction. It has been observed that noradrenergic neurons in the LC are associated with a range of cognitive functions, that hyperactivated noradrenergic neurons lead to working memory impairment, and that excess NE in peripheral and central structures impairs working memory [21]. Animal and human studies have confirmed a dose-dependent “inverted U-shaped” effect of norepinephrine on working memory [22,23], that is, too moderate or too low a norepinephrine phase impairs working memory. For example, a reasonable NE layer on α-2A adrenergic receptors can help enhance working memory, while when the NE layer is too high, norepinephrine will prompt alpha-1 receptors, which may also lead to memory impairment [24]. The results of the present study showed that NE was significantly increased in the hippocampus of chronic sleep deprivation mice compared with the control group (*p* < 0.001), and impaired cognitive and memory abilities were also noted, indicating that the normal mechanism of LC-NE in chronic sleep deprivation mice may also be disturbed, leading to the abnormal release of NE, thereby impairing learning and memory abilities. The abnormal increase in NE may also be related to the excessive excitation of NE neurons, the damage of the “HPA activation-CORT-mediated” neural pathway, and the abnormal expansion of DA. Compared with the model group, norepinephrine (Figure 3B) was significantly reduced in the low-dose *W. coagulans* BC99 group (*p* < 0.05), while norepinephrine was reduced considerably in the high-dose *W. coagulans* BC99 group and the administration group (both *p* < 0.001). These results suggest that *W. coagulans* BC99 may improve cognitive dysfunction induced by chronic sleep deprivation by reducing hippocampal NE content.

At present, a large number of studies have shown that 5-HT can affect learning and memory ability. For example, in rats with vascular dementia, increased levels of 5-HT have been found to improve learning and memory. In addition, some studies have found that 5-HT concentration can maintain or improve memory ability, while decreased concentration levels can impair spatial memory [25]. The results of this study showed that the level of 5-HT (Figure 3C) in the hippocampus of chronic sleep-deprivation mice was lower than that of normal mice, and the difference was statistically significant (*p* < 0.05). The learning and memory ability of chronic sleep deprivation mice decreased, indicating that chronic sleep deprivation reduced 5-HT content in the body and impaired cognition. The content of 5-HT in the hippocampus of chronic sleep deprivation mice was significantly increased in the medium-dose group (up 68.37%) and the piracetam group (all *p* < 0.001), and the high-dose group could significantly increase the content of 5-HT in the hippocampus of chronic sleep deprivation mice (*p* < 0.01) and improve the learning and memory ability of chronic sleep deprivation mice. These results suggest that *W. coagulans* BC99 may improve learning and memory ability by increasing the level of 5-HT in the hippocampus, which may be related to the improvement of DA and 5-HT balance.

Studies on cognition have shown that learning and memory are related to synaptic plasticity in the central nervous system and a large number of chemicals in the brain, such as glutamate, acetylcholinergic, serotonin, and dopamine (DA) [26]. Dopamine (DA) and dopamine D1 receptor (D1R) have been implicated in cognition, cognitive memory, and reward [27]. Among them, DA in the hippocampal DG region plays a regulatory role in synaptic plasticity and analytical memory formation, and this regulatory role is usually achieved through D1R. Studies have found that an appropriate level of DA and D1R activity in the central system can enhance cognitive function and synaptic transmission effects, while excessively high or especially low DA or D1R can damage cognitive aspects and synaptic transmission [28]. For example, large amounts of DA can achieve long-lasting enhancement and improvement of cognitive function by activating D1R [29], while excessive activation of DA-D1R produces neurotoxicity and impairs cognitive function in rats [30], and excessive activation of DA-D1R impairs spatial learning and memory function in rats. The results showed that the information acquisition ability and memory of chronic sleep deprivation mice rats were impaired after chronic sleep deprivation, and the hippocampal DA was abnormally reduced, suggesting that chronic sleep deprivation abnormally reduced the degree of DA release and may no longer activate the DA-D1R system, thereby limiting the ability of learning and recall. On the other hand, whether D1R movement is reduced needs further investigation. In the present study, we found that low-dose BC99 treatment significantly (*p* < 0.05) increased hippocampal DA (Figure 3D) content by 42.02% and improved learning and memory function in chronic sleep deprivation mice, suggesting that BC99 may improve learning and memory potential by increasing hippocampal DA.

Melatonin is widely recognized for its sleep-enhancing properties; however, its role in cognitive impairment caused by insomnia remains unknown, although many animal and scientific studies have shown that melatonin has an enhancing effect on cognitive function [31,32]. Compared with the model group, the MT (Figure 3E) content in the model group was significantly decreased (*p* < 0.01), indicating that chronic sleep deprivation reduced melatonin content in the mice, which resulted in cognitive impairment. The melatonin content of mice in the *W. coagulans* BC99-treated and treated groups did not change significantly compared with the model group. Melatonin can reduce oxidative stress by up-regulating BMAL1, and it can also inhibit microglia activation and NF-κB pathway activation, thereby reducing secondary neuroinflammation, hippocampal neuron damage, and apoptosis, ultimately improving cognitive dysfunction in rats with continuous REM chronic sleep deprivation. Compared with the control group, the blood glucose content (Figure 3F) increased significantly in the model group (*p* < 0.05) and decreased significantly in the piracetam administration group (*p* < 0.05).

### 3.3. Effect of W. coagulans BC99 on Brain and Plasma Inflammation in Chronic Sleep Deprivation Mice

**Figure 4:**
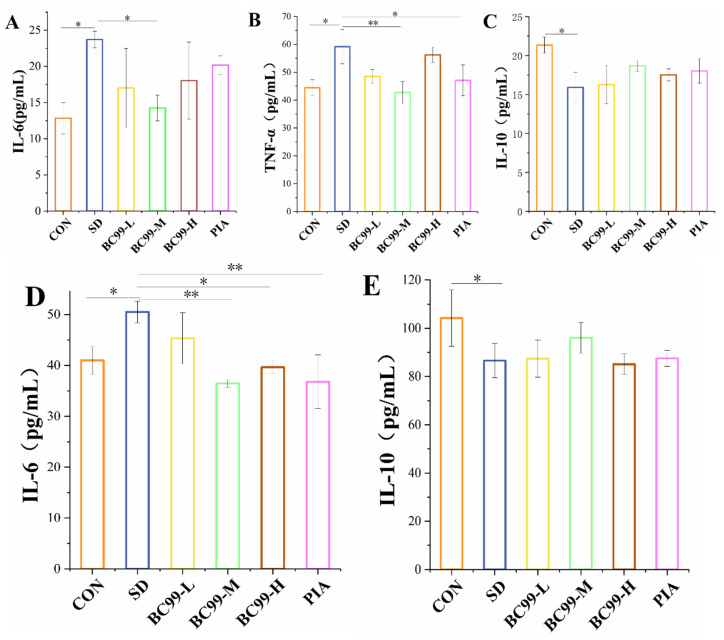
Effect of BC99 on the inflammation of brain and plasma in chronic sleep deprivation mice (n = 3). (**A**) The level of IL-6 in the plasma. (**B**) The level of TNF-α in the plasma. (**C**) The level of IL-10 in the plasma. (**D**) The level of IL-6 in the brain. (**E**) The level of IL-10 in the brain. Note: (**A**,**D**)-interleukin-6; (**B**)-Tumor necrosis factor-α; (**C**,**E**)-interleukin-10. * *p* < 0.05; ** *p* < 0.01.

TNF-α is a pleiotropic pro-inflammatory cytokine that plays diverse roles in regulating a variety of developmental and immune processes, including inflammation, differentiation, lipid metabolism, and apoptosis, and has been implicated in a variety of diseases. In addition, some studies [33] have proved that many pro-oxidative by-products produced by excessive oxidative stress can also stimulate the activity of TNF-α. Conversely, TNF-α can also induce more harmful substances to further aggravate oxidative stress, leading to a vicious circle between excessive oxidative stress and neuroinflammation. TNF-α is a key pro-inflammatory factor that acts synergistically with a variety of cytokines to further induce the release of inflammatory mediators in vivo. IL-10 is an anti-inflammatory factor that inhibits the release of pro-inflammatory factors and reduces inflammatory responses. Figure 3 shows the changes in plasma inflammatory factors in the six groups. Compared with the control group, the levels of IL-6 (Figure 4A) and TNF-α (Figure 4B) in the model group were significantly increased (all *p* < 0.05), and the content of IL-10 (Figure 4C) was significantly decreased (*p* < 0.05), indicating that sleep deprivation could promote the release of inflammatory cytokines. Compared with the model group, the plasma levels of pro-inflammatory cytokines TNF-α and IL-6 (*p* < 0.01) in the *W. coagulans* BC99-protected mice treated with the medium dose were significantly decreased, and the plasma levels of pro-inflammatory factor IL-6 (*p* < 0.05) in *W. coagulans* BC99-protected mice treated with the low dose were significantly decreased. Compared with the model group, the plasma level of TNF-α in the piracetam treatment group was significantly decreased (*p* < 0.05). Compared with the model group, the level of plasma IL-10 in the *W. coagulans* BC99-protected mice was not significantly increased (*p* > 0.05), but the IL-10 level of the high-dose group experienced a greater increase. These results indicate that the medium dose of *W. coagulans* BC99 is more effective in improving inflammation caused by sleep stripping.

It was observed that increased expression levels of the pro-inflammatory cytokines TNF-α, IL-1β, IL-6, and IL-8 decreased expression levels of the anti-inflammatory cytokines IL-4 and IL-10 [34], which is consistent with the experimental results of Zielinski et al. and Manchanda et al. [35,36]. IL-6 has a dual regulatory role in the nervous system: as a neurotrophic factor, it can promote nerve and cell regeneration, while its deficiency may lead to reduced glial activation and changes in sleep and cognitive behavior. Conversely, an excessive increase in IL-6 can contribute to other nervous system pathologies. Figure 4 shows the changes in inflammatory factors in the brain tissue six groups of mice compared with the control group. Compared with the control group, the IL-6 content in the brain tissue of the model group mice was significantly increased (*p* < 0.05) (Figure 4D), and the IL-10 content was significantly decreased (*p* < 0.05) (Figure 4E), indicating that chronic sleep deprivation could induce brain inflammation in mice. Compared with the model group, the level of pro-inflammatory factor IL-6 in the medium-dose group (*p* < 0.01) and high-dose group (*p* < 0.05) protected by *W. coagulans* BC99 significantly decreased. The level of IL-6 in the piracetam group was significantly lower than that in the control group (*p* < 0.01). Medium doses of *W. coagulans* BC99 did not significantly increase the level of plasma IL-10 (10.91%) (*p* > 0.05). These results suggest that *W. coagulans* BC99 at a medium dose is more effective in reducing the inflammatory response induced by chronic sleep deprivation.

### 3.4. Effect of W. coagulans BC99 on Intestinal Barrier in Chronic Sleep Deprivation Mice

**Figure 5:**
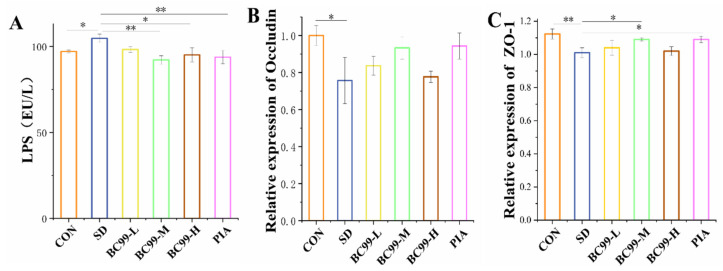
Effect of BC99 on intestinal barrier in chronic sleep deprivation mice. (**A**) The level of LPS in the plasma. (**B**) Relative expression of tight junction protein *Occludin-1* in the jejunum. (**C**) Relative expression of tight junction protein *ZO-1* in the jejunum. * *p* < 0.05, ** *p* < 0.01.

Injury to the intestinal mucosa leads to increased permeability, allowing LPS from Gram-negative bacteria to enter the bloodstream and initiate systemic inflammation. LPS in the plasma (Figure 5A) was significantly higher in the model group than in the control group (*p* < 0.05), indicating that chronic sleep deprivation damaged the intestinal mechanical barrier and increased intestinal permeability in mice with chronic sleep deprivation, which, in turn, led to cognitive impairment. Compared with the model group, the LPS content of the medium-dose BC99 group (*p* < 0.05, 11.99%) and the positive control group (*p* < 0.05, 10.45%) were significantly decreased, indicating that *W. coagulans* BC99 protected mice against intestinal mucosal injury and had no significant effect on intestinal permeability. *W. coagulans* BC99 improved the intestinal mechanical barrier in mice with chronic sleep deprivation, as determined by real-time fluorescence quantitative assay of tight junction protein *ZO-1*. BC99 protects against cognitive impairment induced by chronic sleep deprivation in mice by maintaining the integrity of the intestinal mechanical barrier.

Studies have shown that with the aggravation of intestinal inflammation, intestinal mucosal permeability increases significantly. Intestinal mucosal injury leads to various inflammatory diseases and increased permeability, affecting the intestinal mechanical barrier. Improving intestinal permeability is an effective way to alleviate cognition. In this experiment, LPS concentration and *ZO-1* were used to evaluate the integrity of the intestinal mechanical barrier in mice. Real-time fluorescence quantitative experiments of tight junction proteins *ZO-1* and *Occludin* in mice showed that *W. coagulans* BC99 affected the intestinal mechanical barrier in mice. Chronic sleep deprivation induction significantly reduced the expression of *Occludin* (Figure 5B, *p* < 0.05) and *ZO-1* (Figure 5C, *p* < 0.01) in the jejunal tissue of mice compared with the control group. Low-dose and high-dose *W. coagulans* BC99 treatment could not significantly restore the decreased tight junction protein *Occludin*, while medium-dose *W. coagulans* BC99 treatment could better maintain the integrity of intestinal mucosa, and *Occludin* and *ZO-1* increased by 23.34% and 7.34%, respectively (*p* < 0.05). The destruction of tight junction structure and function in pathological conditions leads to the impairment of intestinal barrier function [37]. The present study demonstrates that *W. coagulans* BC99 protects against cognitive impairment induced by chronic sleep deprivation in mice by maintaining the integrity of the intestinal mechanical barrier.

### 3.5. Effects of BC99 on Intestinal Flora in Chronic Sleep Deprivation Mice

**Figure 6:**
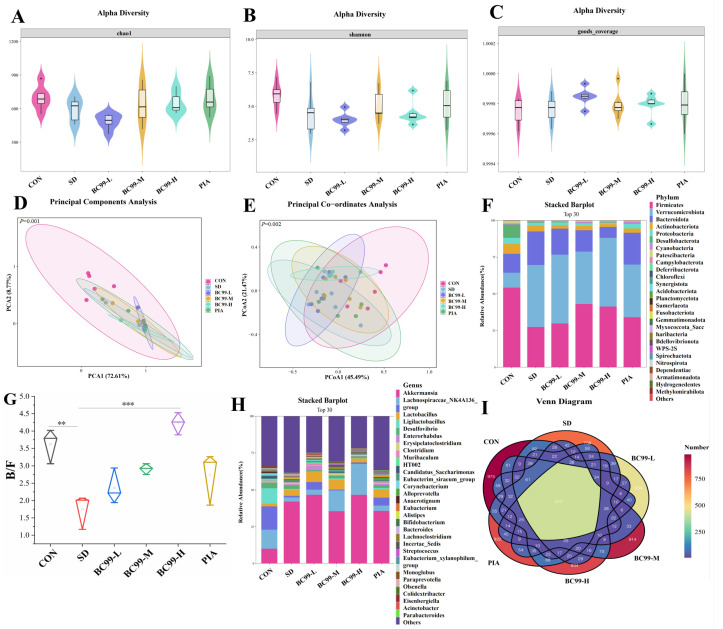
Effects of BC99 on intestinal flora and functional prediction in chronic sleep deprivation mice. α and β diversity in the intestinal flora of mice in each group (**A**–**E**). (**A**) Chao1 index. (**B**) Shannon index. (**C**) Goods_coverage. (**D**) PCA. (**E**) PCOA. Figure 5 Composition of the mouse intestinal flora at the phylum and genus levels in each group (**F**,**G**). (**F**) Column-stacked plot at the phylum level for relative abundance of TOP30. (**G**) Heatmap of relative abundance of TOP30 at the genus level. (**H**) B/F. (**I**) Venn diagram. Note: (**D**)—principal component analysis, (**E**)—principal coordinate analysis, (**H**)—Hyphomycetes/Phyllobacteriophage, and (**I**)—Wayne diagram. ** *p* < 0.01; *** *p* < 0.001.

Chao1 mainly reflects the richness of a community, and Shannon reflects the diversity of a community. Both the Shannon (Figure 6A) and Chao1 (Figure 6B) indices were decreased in the chronic sleep deprivation group (both *p* = 0.09), but these effects were reversed by *W. coagulans* BC99, indicating that *W. coagulans* BC99 could enhance α diversity in chronic sleep deprivation mice. The Goods_coverage (Figure 6C) values of each group were all 1.0, indicating that the sequencing results accurately reflected the actual situation of the samples. *W. coagulans* BC99, especially at medium doses, improved colony diversity to a level comparable to drug therapy. Similarity or difference (β-diversity) in gut microbial community composition was assessed using principal component analysis (PCA) based on Euclidean distance and principal coordinate analysis (PCoA) based on weighted uniFrac distance. PCA (Figure 6D) showed that the intestinal flora of the SD group was significantly different from that of the blank control group (*p* < 0.01), and the intervention of BC99 could improve the abnormal changes in intestinal flora. The weighted analysis showed that the distribution of PCOA (Figure 6E) between the SD group and the normal sleep control group was far apart, and the *p*-value was <0.01, which was a significant statistical difference, indicating that SD significantly reduced the β diversity of intestinal microorganisms. Medium-dose BC99 treatment reduced the distance between gut microbiota, indicating that this significant species difference was restored to the same extent as drug treatment. It should be noted that the PCA and PCOA analyses themselves were calculated without *p*-values, which were derived from the results of the ANOSIM analysis. We added a *p*-value to the PCOA plot when plotting. These results suggested that BC99 ameliorated cognitive impairment by increasing the diversity and narrowing the difference in gut microbiota composition in SD mice.

At the phylum (Figure 6F) level, *Firmicutes* and *Bacteroidetes* were dominant in all samples, with a combined percentage of 80 to 90%. At the genus level (Figure 6G), regarding *Lachnospiraceae_NK4A136_group*, *Lactobacillus*, *Desulfovibrio*, *Enterorhabdus*, *HT002*, *Anaerotignum*, Akkermansia, the abundance of *Corynebacterium* and *Muribaculum* was higher. *Lactobacillus* can secrete antibiotics to antagonize pathogenic bacteria. GABA levels are found to be reduced in the frontal, temporal, and parietal cortex of AD patients, while certain *Bifidobacteria* and *Lactobacillus* in the gut may convert glutamate into GABA to regulate the body’s cognitive behavior. *Blautia* and *Pilospirillum* can produce a large number of SCFAs and promote intestinal health. SCFAs are involved in DA and NE synthesis and have modulatory effects on serotonergic neurotransmission and lower levels of GABA, serum amine, and DA in the body. In this experiment, the abundance of *Lactobacillus*, *Lachnospiraceae_NK4A136_group*, and *Desulfovibrio* decreased in the model group. The abundance of these beneficial bacteria increased significantly in all of the BC99 intervention groups, at all doses, and the decrease was more pronounced in the medium- and high-dose groups, suggesting that BC99 may be related to the effective alleviation of cognitive impairment caused by chronic sleep deprivation. *Alistipes* and *Olsenella* increased in the SD group, while the abundance of harmful bacteria decreased significantly in all of the BC99 intervention groups, at all doses, especially in the medium- and high-dose groups, indicating that medium and high doses of BC99 could effectively inhibit the growth of harmful bacteria, maintaining the normal structure of intestinal flora. As a representative of the Bacteroidetes phylum, the relative abundance of the other genera is higher in stressed quail with impaired spatial memory. The harmful effects of the other genera may be related to the intestinal permeability caused by the imbalance of intestinal flora, which can increase the levels of harmful substances such as lipopolysaccharide in the blood, leading to neuroinflammation and behavioral changes. Regarding the increase in the genus *Akkermansia muciniphila* in this experiment, it may be due to the large number of BC99 consuming the free oxygen in the gut, providing it with a better anaerobic environment for growth. These results indicate that administering BC99 at medium and high doses can effectively increase beneficial bacteria’s growth and maintain intestinal flora’s normal structure.

Studies have found that the ratio of *Firmicutes* to *Bacteroidetes* (F/B) can be used to evaluate the intestinal pathological condition of the body, and the imbalance of the ratio of *Firmicutes* to *Bacteroidetes* may lead to various metabolic diseases. The results of this experiment showed that the F/B (Figure 6H) value of the SD group was 1.75, which was significantly lower than the 3.62 of the CON group (*p* < 0.01), indicating that sleep deprivation had a significant effect on the structure of intestinal flora. The F/B values of the SD-LC, SD-MC, SD-HC, and SD-HC groups were 2.36, 2.91, 4.23, and 2.74, which were 1.35, 1.66, 4.62, and 2.42 times that of SD group, respectively. These results suggest that coagulated *W. coagulans* BC99 supplementation plays a positive role in regulating the structure of gut microbiota in mice during 4 weeks of sleep deprivation. This result is consistent with many previous animal and clinical studies. The Venn diagram in Figure 6I shows a total of 417 species across the six treatment groups.

### 3.6. Differential Effects of W. coagulans BC99 on LEfSe and Prediction of PICRUSt2 Function in Chronic Sleep Deprivation Mice

**Figure 7:**
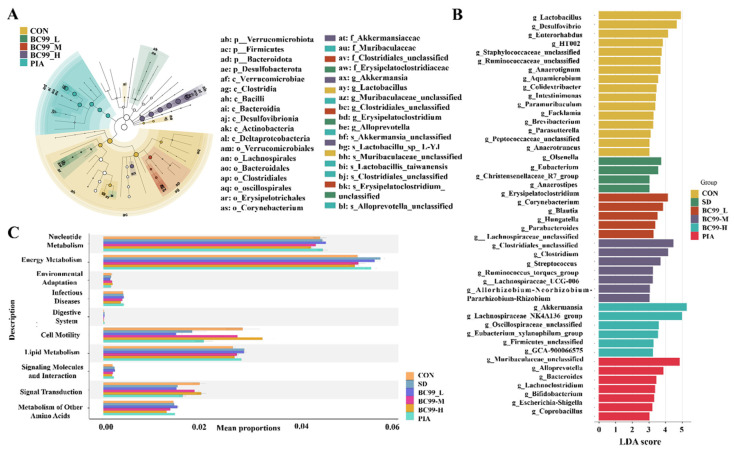
Differential effects of BC99 on LEfSe and prediction of PICRUSt2 function in chronic sleep deprivation mice. (**A**–**C**): (**A**) Evolutionary branching diagram. (**B**) Histogram of LDA distribution. (**C**) PICRUSt2 function prediction.

Figure 7A,B, combined with the genus-level heatmap (Figure 6G), shows that the expression of Euromonas, Anaerostipes, and Christensenellaceae_R7_group was significantly up-regulated in the model group compared to the control group. The abundance of Trichosporon and *Eubacterium_xylanophilum_group* was significantly up-regulated in the *W. coagulans* BC99 high-dose group. *Tricoron* sp. hydrolyzes starch and other sugars to produce butyrate and other short-chain fatty acids, which regulate intestinal inflammation by promoting integrity and immune responses. SCFAs may improve intestinal epithelial resistance by up-regulating the levels of tight junction proteins and mucin. Fecal butyric acid levels were positively correlated with *Lachnospiraceae_NK4A136_group* and *Eubacterium_xylanophilum_group*. Sodium butyrate significantly improves learning and memory in AD models [38,39]. Anaerostipes of Firmicutes was significantly increased in the PSCI group and was negatively correlated with MoCA, suggesting that it was associated with cognitive decline. These results indicated that *W. coagulans* BC99 intervention reduced gut pathogenic bacteria, increased probiotics, maintained intestinal homeostasis, and alleviated chronic sleep deprivation in mice. KEGG Level3 PICRUSt2 (Figure 7C) function prediction showed that chronic sleep deprivation was related to nucleotide metabolism, energy metabolism, environmental adaptation, infectious diseases, the digestive system, cell activity, lipid metabolism, and signal molecules and their interactions rather than chronic sleep deprivation action and amino acid metabolism.

### 3.7. Effects of W. coagulans BC99 on Short-Chain Fatty Acids in Chronic Sleep Deprivation Mice

Short-chain fatty acids (SCFAs), as gut microbial metabolites, play an important role in the brain–gut axis. Acetic acid, propionic acid, and butyric acid are key neuromediators that can cross the blood–brain barrier (BBB) to affect brain-related functions and participate in the biosynthesis of neurotransmitters and neurotrophic factors [13]. Notably, butyrate enhanced tight junction protein expression and increased BBB integrity in mice [40] while also regulating cell autophagy and increasing α-synuclein RNA expression through the PI3K/Akt/mTOR signaling pathway [41]. As endogenous ligands of orphan G protein-coupled receptors (GPCRs), SCFAs directly affect gene expression by inhibiting histone deacetylation (HDAC) [13] and then regulate the differentiation, recruitment, and activation of various immune cells [42]. In one study, acetate, butyrate, and propionate were shown to reduce the levels of IL-6 and IL-8 in HUVEC cells after LPS or TNF-α stimulation, and their effects were time-dependent [43]. In addition, SCFAs may reduce bacterial translocation through the protective effect of intestinal barrier function, thereby reducing systemic inflammatory response and indirectly reducing neuroinflammation in the brain [44].

**Figure 8:**
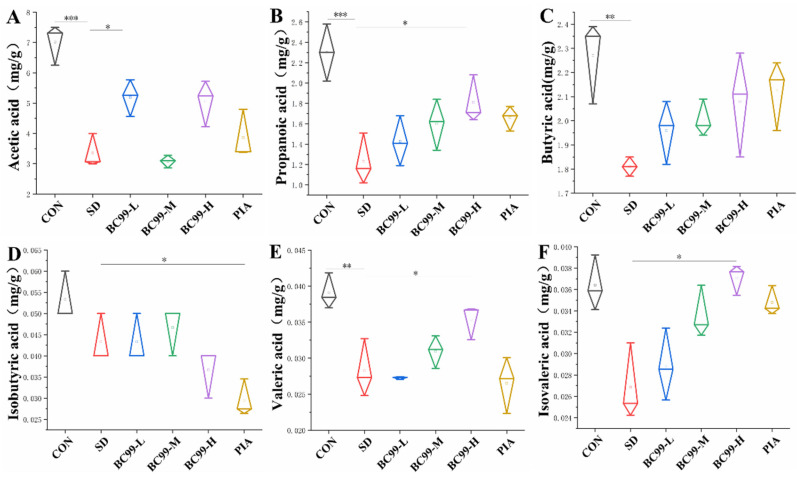
Effects of BC99 on short-chain fatty acids and in chronic sleep deprivation mice. (**A**) The level of acetic acid in the feces. (**B**) The level of propionic acid in the feces. (**C**) The level of butyric acid in the feces. (**D**) The level of isobutyric acid in the feces. (**E**) The level of valeric acid in the feces. (**F**) The level of isovaleric acid in the feces. * *p* < 0.05, ** *p* < 0.01, and *** *p* < 0.001.

To elucidate the effect of *W. coagulans* BC99 on metabolites of gut microbiota, SCFAs content in mouse feces was measured in this experiment, and the results are shown in Figure 8. The fecal levels of propionic acid (Figure 8B, *p* < 0.01), butyric acid (Figure 8C, *p* < 0.05), and valeric acid (Figure 8E, *p* < 0.01) were significantly lower in the model group than in the control group, and the acetic acid level (Figure 8A) was significantly lower (*p* < 0.001). Isovaleric acid (Figure 8D) and isobutyric acid (Figure 8F) levels decreased but not significantly (*p* > 0.05). Compared with the model group, the content of acetic acid in the low-dose BC99 group was significantly increased (54.66%, *p* < 0.05), and the content of isobutyric acid in the high-dose *W. coagulans* BC99 group was significantly increased (*p* < 0.05); the high dose of propionic acid led to an increase of 47.15%, and the high dose of butyric acid led to an increase of 14.92%, and the increases in valeric acid and isovaleric acid were not significant (*p* > 0.05). Isovaleric acid was significantly decreased in the medication group (*p* < 0.05). These results indicated that both low and high doses of *W. coagulans* BC99 reversed the decreasing trend of SCFAs content in the feces of chronic sleep-deprived mice.

### 3.8. Effect of W. coagulans BC99 on NLRP3 Inflammasome Signaling Pathway in the Brain and Jejunum of Chronic Sleep Deprivation Mice

**Figure 9:**
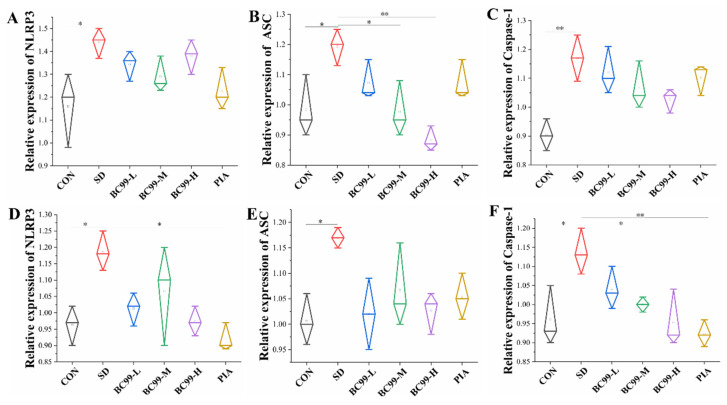
Effect of BC99 on *NLRP3/ASC* inflammasome signaling pathway in the brain and jejunum of chronic sleep-deprived mice**.** (**A**–**F**). (**A**) Relative expression of *NLRP3* in the jejunum. (**B**) Relative expression of *ASC* in the jejunum. (**C**) Relative expression of *Caspase-1* in the jejunum. (**D**) Relative expression of *NLRP3* in the brain. (**E**) Relative expression of *ASC* in the brain. (**F**) Relative expression of *Caspase-1* in the brain. Note: (**A**)—crypto thermal protein, (**B**)—apoptosis-associated speck-like protein, and (**C**)—cysteinyl aspartate specific proteinase-1. * *p* < 0.05, ** *p* < 0.01.

The *NLRP3* inflammasome has been implicated in a variety of diseases, including gout, type 2 diabetes, and Alzheimer’s disease [45]. Alzheimer’s disease is a neurodegenerative disease characterized by progressive memory loss and cognitive dysfunction [46]. The *NLRP3* inflammasome has played a key role in cognitive abnormalities and may also be a viable therapeutic target [47,48]. The relative expression levels of *NLRP3* (*p* < 0.05) (Figure 9A), *ASC* (*p* < 0.05) (Figure 9B), and *Caspase-1* (*p*< 0.05) (Figure 9C) in the jejunum of the model group were significantly increased, indicating that intestinal inflammation was increased in mice with cognitive impairment induced by sleep deprivation. *W. coagulans* BC99 treatment with medium (*p* < 0.05) (decreased by 17.65%) and high (*p* < 0.01) (decreased by 26.05%) doses significantly reduced the relative expression of *ASC*, and *W. coagulans* BC99 treatment significantly reduced intestinal inflammation in mice with cognitive impairment induced by sleep deprivation. These results indicated that intestinal inflammation in mice was ameliorated by treatment with *W. coagulans* BC99. This study suggests that *W. coagulans* BC99 indirectly improves cognitive impairment by inhibiting the expression of *NLRP3/ASC* signaling in the intestinal tract of SD mice. In the model, the relative expression levels of *NLRP3* (Figure 9D) and *ASC* (Figure 9E) were significantly higher in the model group (*p* < 0.05), which was 12.82%. After high-dose *W. coagulans* BC99 treatment, the relative expression of Caspase-1 (Figure 9F) in the model group was significantly increased (*p* < 0.05), and the relative expression of Caspase-1 in the positive control group (*p* < 0.01) was significantly decreased (by 12.28%) (*p* < 0.05). These results indicate that the inflammatory response was attenuated in the mouse brain after treatment with *W. coagulans* BC99. *W. coagulans* BC99 may alleviate cognitive dysfunction in sleep-deprived mice by inhibiting the activation of the *NLRP3/ASC* signaling pathway.

**Figure 10:**
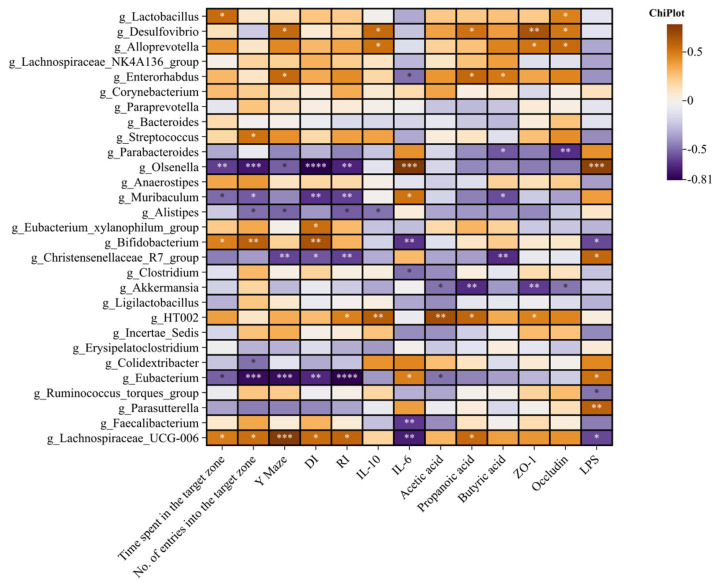
Spearman correlation analysis was used to analyze the correlation between gut microbiota and behavioral tests, SCFAs, brain inflammatory factors, intestinal tight junction proteins, and LPS. * *p* < 0.05, ** *p* < 0.01, *** *p* < 0.001, **** *p* < 0.0001.

Spearman correlation analysis was used to analyze the correlation between gut microbiota and behavioral tests, SCFAs, brain inflammatory factors, intestinal tight junction proteins, and LPS. The results are shown in Figure 10. below. *Lactobacillus* was significantly positively correlated with the time of crossing the target quadrant, and *Streptococcus* was significantly positively correlated with the number of target quadrant crossings. There was a significant negative correlation between the *Muribaculum* and the times for crossing the target quadrant, time, cognitive index, discrimination index, and propionic acid and a positive correlation between the *Muribaculum* and brain pro-inflammatory factor IL-6, indicating that the *Muribaculum* shows the characteristics of promoting cognitive impairment. *Lachnospiraceae_UCG_006* and *Bifidobacterium* were significantly positively correlated with the resolution index, the number, and the time of crossing the target quadrant of the water maze. *Desulfvibrio*, *Enterorhabdus*, and *Lachnospiraceae_UCG_006* were significantly positively correlated with the Y-maze new arm time. *Olsenella* and *Eubacterium* were negatively correlated with the time, discrimination index, cognitive index, and the number and time of target quadrant crossing in the Morris water maze experiments. *Christensenelaceae_R7_group* was negatively correlated with the time, discrimination index, and cognitive index of the new arm of the Y maze. *Eubacterium_xylanophilum_group* was positively correlated with the resolution index. *Lachnospiraceae_NK4A136_group*, *Enterorhabdus*, *Lachnospiraceae_UCG_006*, *Eubacterium_xylanophilum_group*, *HT002*, *Bifidobacterium*, *Lactobacillus*, and *Streptococcus* showed the characteristics of alleviating cognitive impairment, while *Christensenelaceae_R7_group*, *Olsenella*, and *Eubacterium* showed the characteristics of causing cognitive impairment.

*Desulfvibrio*, *Alloprevotella*, and *HT002* were significantly positively correlated with brain anti-inflammatory factors, which showed the characteristics of alleviating inflammation. The IL-10 in the brain was significantly negatively correlated with *Alistipes*, which showed the characteristics of promoting inflammation. *Olsenella*, *Muribaculum*, and *Eubacterium* were significantly positively correlated with pro-inflammatory factors in the brain, showing the characteristics of promoting inflammation. *Bifidobacterium*, *Clostridium*, *Faecalibacterium*, and *Lachnospiraceae_UCG_006* were significantly negatively correlated with pro-inflammatory factors in the brain, showing the characteristics of alleviating inflammation.

*Desulfurvibrio*, *Enterorhabdus*, *Lachnospiraceae_UCG_006*, and *HT002* were positively correlated with acetic acid, propionic acid, and butyric acid, and their relative abundance was reduced in the model group. This suggests that they are positively correlated with an increase in SCFAs. LPS was positively correlated with *Alistipes*, *Christensenelaceae_R7_group*, *Eubacterium*, and *Parasutterella*. LPS was significantly negatively correlated with *Bifidobacterium*, *Ruminococcus_torques_group*, and *Lachnospiraceae_UCG_006*. The relative abundance of *Desulfurvibrio* and *Alloprevotella* in the model group decreased and positively correlated with *ZO-1* and *Occludin*, indicating that they exhibited the characteristics of enhancing the intestinal barrier.

## 4. Discussion

**Figure 11:**
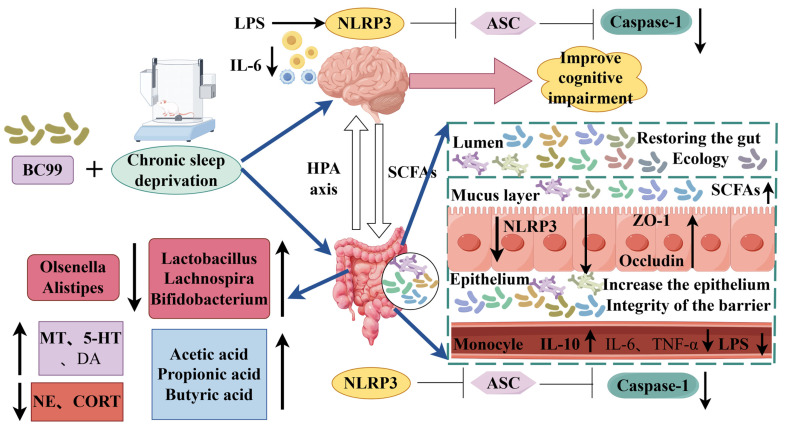
Diagram of the mechanism by which *Weizmannia coagulans* BC99 improves cognitive impairment in chronic sleep-deprived mice.

Chronic sleep deprivation is becoming increasingly common in modern society. Prior research by Wang et al. [49] has established that chronic sleep deprivation can significantly alter the human gut microbiota, which subsequently contributes to cognitive impairments associated with chronic sleep deprivation. The current study aims to examine the effects of *W. coagulans* BC99 on neurological issues resulting from chronic sleep deprivation, investigating potential mechanisms through the framework of the microbe–gut–brain axis (Figure 11). Behavioral assessments indicated that chronic sleep deprivation has a pronounced negative impact on cognitive functions, particularly in learning and memory. Morris water maze experiments demonstrated an increase in latency to find the platform and a reduction in both the time spent and the number of crossings into the target quadrant among rodents subjected to chronic sleep deprivation. The cognitive deficits identified in the novel object recognition (NOR) test corroborate previous findings [50,51], and treatment with *W. coagulans* BC99 effectively ameliorated these behavioral deficits. The administration of *W. coagulans* BC99 appears to provide a protective effect against cognitive impairments induced by chronic sleep deprivation, with its mechanism of action associated with the neuroendocrine system. The hypothalamus is integral to the regulation of circadian rhythms and the hormonal secretion of the hypothalamic–pituitary–adrenal (HPA) axis. Increased plasma levels of adrenocorticotropic hormone (ACTH) and cortisol (CORT) are thought to result from HPA axis activation [52,53], which is alleviated by *W. coagulans* BC99 treatment, leading to a reduction in plasma CORT levels. Furthermore, serotonin (5-HT) and melatonin are essential for promoting sleep and regulating sleep/wake cycles. Following *W. coagulans* BC99 treatment, an increase in hippocampal 5-HT levels was observed, suggesting that *W. coagulans* BC99 enhances sleep and subsequently improves cognitive function through the elevation of brain 5-HT.

Additionally, gut microbiota may represent a potential etiological factor in the inflammation and cognitive impairments associated with chronic sleep deprivation. Changes in gut microbiota composition have been linked to the progression of Alzheimer’s disease pathology. Research utilizing animal models has shown that the proportion of the thick-walled phylum/Anthrobacter phylum is reduced in Alzheimer’s disease (AD) mice compared to wild-type (WT) mice, with significant alterations noted in the levels of both families and genera of butyrate-producing bacteria. Notably, the levels of a specific genus within the Trichosporon family have been observed to increase as the disease advances in AD mice [9,54]. Our study indicates that chronic sleep deprivation results in a decrease in gut microbiota diversity in mice, leading to changes in gut flora composition. After four weeks of chronic sleep deprivation, a significant reduction in the relative abundance of the thick-walled phylum was noted, while an increase in the relative abundance of the anapestic phylum was observed. At the genus level, there was a decline in the relative abundance of short-chain fatty acid (SCFA)-producing bacteria, including *Lactobacillus* spp., *Bifidobacterium* spp., and *Trichoderma* spp., alongside an increase in the relative abundance of the pathogenic bacterium *Eubacterium* spp. Currently, the activation of the inflammatory response is considered a potential mechanism that links sleep disorders to an increased risk of AD.

Various experimental methodologies, such as complete nocturnal chronic sleep deprivation, chronic sleep restriction, and partial nocturnal chronic sleep deprivation, have been shown to activate inflammatory markers. Notably, these include the activation of the NF-κB signaling pathway, the production of pro-inflammatory cytokines, and an increase in systemic inflammation, as evidenced by elevated levels of C-reactive protein (CRP). A meta-analysis encompassing 72 studies with over 50,000 participants revealed a significant association between sleep disorders and heightened levels of peripheral blood inflammation. Furthermore, concentrations of inflammatory markers such as CRP and interleukin-6 (IL-6) were positively correlated with the severity of sleep disturbances. Elevated peripheral inflammatory factors may trigger the activation of central microglia, leading to central nervous system (CNS) inflammation, which is implicated in cognitive decline and an increased risk of developing Alzheimer’s disease (AD). Clinical investigations have demonstrated that levels of pro-inflammatory cytokines in the plasma and cerebrospinal fluid of patients with AD and mild cognitive impairment (MCI) progressively increase throughout the disease’s progression, indicating that systemic inflammation may exacerbate cognitive decline. *NLRP3* inflammasomes, as critical components of the innate immune system, play a significant role in the body’s immune response and the progression of various diseases. Their activation, which can be triggered by a wide array of pathogens, is pivotal in numerous pathological processes, leading to the downstream release of IL-1β and IL-18, which are involved in the regulation of various diseases. The activation of *NLRP3* inflammasomes in the brain is associated with the modulation of synaptic and microglial cell functions, as well as the activation of immune-inflammatory responses, all of which can influence cognitive function. An animal study indicated that the deficiency of *NLRP3* in the hippocampus of AD transgenic mice improved their spatial memory capabilities by mitigating inflammatory responses and microglial activation in the brain. Additionally, another study found that aggregates of β-amyloid and tau proteins activated *NLRP3* inflammasome signaling in the brain, contributing to both pathophysiological changes and cognitive decline in AD [55]. Research has demonstrated that the activation of the *NLRP3* inflammasome occurs within the gastrointestinal tract and that gut microbiota can influence brain function via the gut–brain axis by modulating the *NLRP3* inflammatory pathway [56]. In the present study, we observed that alterations in gut microbiota serve as a contributing factor to chronic sleep deprivation, which results in the compromise of the intestinal barrier. This disruption leads to the release of lipopolysaccharides (LPSs), which, upon entering the central nervous system (CNS) through a compromised blood–brain barrier, activate *NLRP3* inflammatory vesicles. This activation is associated with an increase in the inflammatory cytokine IL-6 within the murine brain.

In summary, *W. coagulans* BC99 may influence intestinal barrier integrity and inflammatory responses by modulating gut microbiota, thereby mitigating CNS inflammation and cognitive deficits associated with chronic sleep deprivation. These findings provide a scientific foundation for further exploration of *W. coagulans* BC99’s potential in enhancing cognitive function. However, it is important to note that this study offers preliminary evidence regarding the modulatory effects of *W. coagulans* BC99 on cognitive impairment in sleep-deprived mice, with its limitations including the small sample size and a primary focus on animal models, as well as the fact that this study was an exclusive investigation of gut microbiota without a comprehensive analysis of metabolites. Consequently, additional clinical studies confirm its efficacy and safety in human populations. Future research could further investigate the effects of *W. coagulans* BC99 on various cognitive disorders and its potential synergistic effects when combined with other therapeutic approaches (e.g., pharmacotherapy, and behavioral interventions). The potential of *W. coagulans* BC99 as a probiotic for ameliorating cognitive impairment induced by chronic sleep deprivation merits further examination. Future studies should also consider the application of *W. coagulans* BC99 in human sleep disorders and cognitive decline, as well as identify the key metabolites involved in these processes.

## 5. Conclusions

In this study, we discovered that chronic sleep deprivation causes alterations in the gut microbiota of mice, which play a crucial role in mediating the cognitive decline associated with chronic sleep deprivation. This cognitive decline further impaired the intestinal barrier and immune inflammatory response in the brain, negatively affecting cognitive function. *NLRP3* inflammatory vesicles were identified as a key component of the observed cognitive impairment reversed by *W. coagulans* BC99 administration in mice. Our results suggest that *W. coagulans* BC99 supplementation attenuates cognitive impairment caused by chronic sleep deprivation. The mechanism involves *W. coagulans* BC99 up-regulating the expression of jejunal tight junction proteins, which is conducive to repairing the intestinal barrier. *W. coagulans* BC99 inhibits the activation of the *NLRP3/ASC* inflammasome signaling pathway in the jejunum, which can reduce LPS and related inflammatory factors in peripheral circulation and inhibit the activation of the *NLRP3/ASC* inflammasome signaling pathway in the brain; these factors can reduce the inflammatory response and ultimately improve cognitive impairment caused by SD. These findings lay the foundation for potential clinical strategies for managing insomnia and insomnia-induced cognitive impairment through dietary interventions.

## Figures and Tables

**Table 1:** Primer sequences.

Gene	Primer Sequence
*Occludin*	F: CTCGGTACAGCAGCAATGGT R: TCATAGTGGTCAGGGTCCGT
*ZO-1*	F: ATTCAGGTCGCTCGCATGAC R: ACTGCGTGGAATGATCGGAG
*NLRP3*	F: CCAGGAGTTCTTTGCGGCTA R: GCCTTTTTCGAACTTGCCGT
*ASC*	F: AGACCACCAGCCAAGACAAG R: CTCCAGGTCCATCACCAAGT
*Caspase-1*	F: AACCACTCGTACACGTCTTGCC R: CCAGATCCTCCAGCAGCAACTT
*β-Actin*	F: CTGTGTTTTGGTCTTACGGTAC R: AAAAAGCCTGTCTGTGATTCAC

## Data Availability

The original contributions of this study are included in the article; further inquiries can be directed to the corresponding authors.
